# QTc Interval Prolongation and Associated Drug Therapy in Hospitalized Older Adults

**DOI:** 10.1111/jgs.70477

**Published:** 2026-05-02

**Authors:** Francesco Fantin, Sara Fiorini, Anna Giani, Luana Giacobbe, Elena Zoico, Gianluca Trifirò, Rocco Micciolo, Mauro Zamboni

**Affiliations:** ^1^ Centre for Medical Sciences—CISMed, Department of Psychology and Cognitive Science University of Trento Rovereto Italy; ^2^ Department of Medicine, Section of Geriatrics University of Verona Verona Italy; ^3^ Department of Diagnostics and Public Health University of Verona Verona Italy; ^4^ CISMed University of Trento Trento Italy

**Keywords:** drug‐induced arrhythmias, geriatric patients, polypharmacy, QTc prolongation, Tisdale score

## Abstract

- Key points
  ○ QTc interval prolongation affects nearly one‐third of hospitalized older adults, with 15.8% reaching the high‐risk threshold (> 500 ms).
  ○ Polypharmacy with QTc‐prolonging drugs and diuretics is highly prevalent and represents a major, potentially modifiable risk factor.
  ○ The Tisdale risk score reliably stratifies the risk of severe QTc prolongation in geriatric inpatients, even after adjustment for multimorbidity and renal dysfunction.
- Why does this matter?
  ○ QTc prolongation is frequent yet under‐recognized in hospitalized older adults, exposing them to preventable life‐threatening arrhythmias. Routine use of the Tisdale score at admission can guide medication review and cardiac monitoring in geriatric practice.

## 1 Introduction

QTc interval prolongation predisposes to torsades de pointes (TdP) and sudden cardiac death [1]. Older adults are particularly vulnerable due to polypharmacy, multimorbidity, age‐related pharmacokinetic, and pharmacodynamic changes, and electrolyte disturbances [2]. Multiple drug classes commonly prescribed to older patients prolong the QTc interval through hERG channel blockade, including antiarrhythmics, antipsychotics, antidepressants, and proton pump inhibitors (PPIs) [1, 2]. Observational studies report QTc prolongation in 27%–37% of older adults at hospital admission [3, 4], yet systematic risk stratification remains underutilized in geriatric practice. The Tisdale risk score is a validated tool for identifying hospitalized patients at risk of drug‐induced QTc prolongation [5], but its performance in exclusively geriatric populations has not been studied. We evaluated QTc prevalence, medication profiles, and Tisdale score performance in a real‐world acute geriatric cohort.

## 2 Methods

This cross‐sectional study enrolled 494 consecutive older adults (≥ 65 years; mean age 84.5 ± 6.9 years; 53.2% male) admitted to the acute geriatric ward of the University of Verona between September 2018 and April 2019. Twelve‐lead ECG was obtained at admission with automated QTc calculation, independently validated by a cardiologist. QTc prolongation was defined as > 470 ms in males and > 480 ms in females; severe prolongation as > 500 ms [1]. Comorbidity was quantified by the Charlson Comorbidity Index [6] and renal function by CKD‐EPI eGFR. Medications were classified as QTc‐prolonging per CredibleMeds criteria. Tisdale risk scores were calculated and patients stratified as low‐ (≤ 6), intermediate‐ (7–10), or high‐risk (≥ 11) [5]. Logistic regression assessed the association between Tisdale score and QTc > 500 ms before and after adjustment for Charlson Index, eGFR, electrolytes, and diuretic use. The study was approved by the Ethics Committee of Verona and Rovigo (protocol 1892CESC).

## 3 Results

Baseline characteristics are shown in Table 1. QTc prolongation was detected in 150 patients (30.4%); 78 (15.8%) had severely prolonged QTc (> 500 ms), associated with markedly increased TdP risk [1]. No significant sex differences were observed in QTc values (males: 459.2 ± 42.6 ms vs. females: 459.6 ± 42.2 ms; *p* = 0.908) or prevalence (30.8% vs. 29.9%) Figure S1.

**TABLE 1:** Baseline characteristics of the study population.

Variable	Males (*n* = 263)	Females (*n* = 231)	Total (*n* = 494)	*p*
Age (years)	83.40 ± 6.96	85.12 ± 6.65	84.21 ± 6.86	0.005
Weight (kg)	71.42 ± 14.51	63.81 ± 13.89	67.82 ± 14.70	< 0.001
Charlson Index	6.04 ± 2.14	5.90 ± 1.88	5.98 ± 2.02	0.436
eGFR (ml/min)	59.55 ± 23.21	57.25 ± 22.84	58.47 ± 23.04	0.268
Sodium (mmol/L)	139.57 ± 4.41	140.60 ± 5.21	140.05 ± 4.82	0.019
Potassium (mmol/L)	3.92 ± 0.56	3.72 ± 0.53	3.82 ± 0.56	< 0.001
QTc (ms)	459.17 ± 42.61	459.61 ± 42.17	459.38 ± 42.36	0.908
Tisdale score	5.64 ± 3.32	7.31 ± 3.53	6.42 ± 3.52	< 0.001

*Note:* Data presented as mean ± SD.

Abbreviations: eGFR, estimated Glomerular Filtration Rate (CKD‐EPI); SD, Standard Deviation.

**FIGURE 1:**
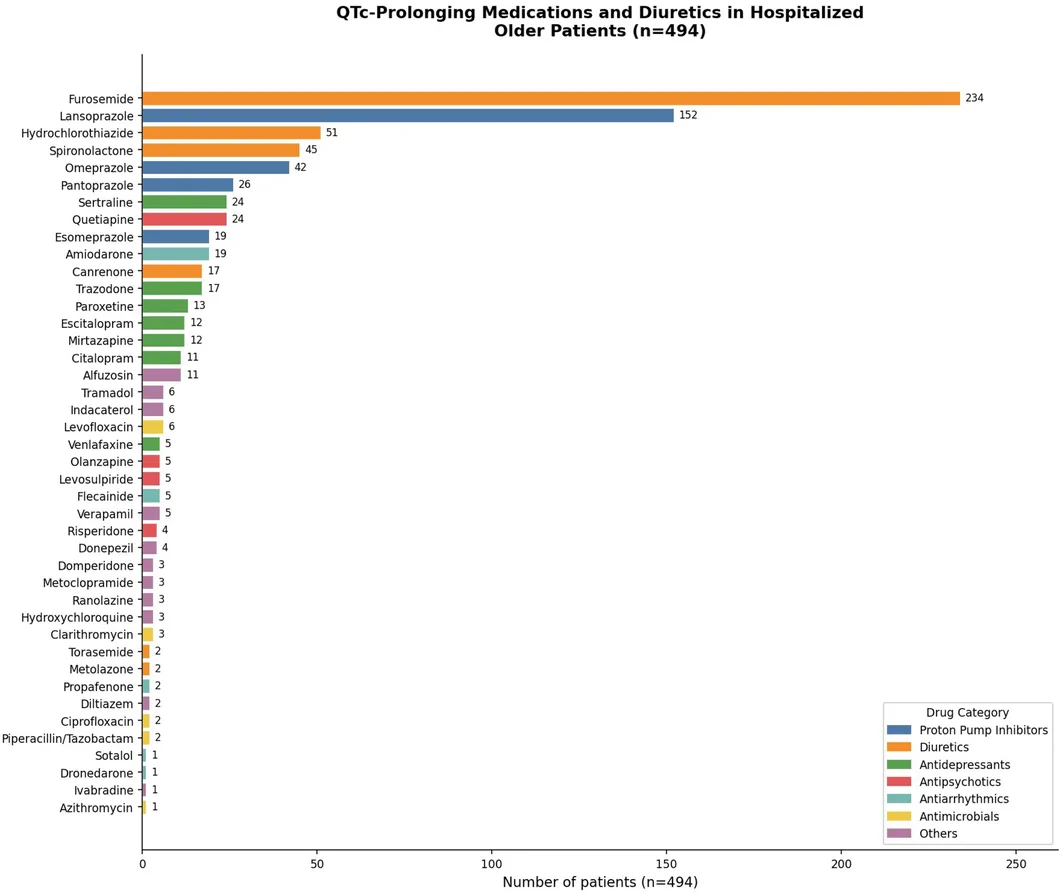
QTc‐Prolonging Medications and Diuretics in Hospitalized Older Adults (*n* = 494) Bars represent the number of patients on chronic therapy with each drug at admission. Color coding by drug category: Blue = proton pump inhibitors; orange = diuretics; green = antidepressants; red = antipsychotics; teal = antiarrhythmics; yellow = antimicrobials; purple = other agents. SSRIs = selective serotonin reuptake inhibitors.

Figure 1 shows the medication profile. PPIs were the most frequently prescribed QTc‐prolonging class (48.4%), predominantly lansoprazole (*n* = 152). Antidepressants were used by 94 patients (19.0%), primarily SSRIs. Antiarrhythmics and antipsychotics were each prescribed to 33 patients (6.7%). Loop diuretics were prescribed to 236 patients (47.8%), with diuretic‐induced electrolyte disturbances—particularly hypokalemia—further amplifying QTc risk.

Tisdale stratification classified 53.0% as low‐risk, 33.8% as intermediate‐risk, and 13.2% as high‐risk [5]. Scores were significantly higher in females (7.31 ± 3.53 vs. 5.64 ± 3.32; *p* < 0.001). A significant positive correlation was found between Tisdale score and QTc (Pearson *r* = 0.34; *p* < 0.001). Logistic regression showed a clear dose–response relationship: Predicted probability of QTc > 500 ms was 5.4% at score 0, ~26% at score 10, and exceeded 50% at score 17 (unadjusted OR per 1‐point increase: 1.19, 95% CI 1.13–1.26; *p* < 0.001; Figure S2). This association persisted after full adjustment (OR = 1.14, 95% CI 1.07–1.21; *p* < 0.001).

## 4 Discussion

Our findings confirm that QTc prolongation affects nearly one‐third of hospitalized older adults, consistent with a prior Italian study reporting 36.6% prevalence in an internal medicine population [3] and a Serbian geriatric cohort reporting 27.3% at admission [4]. The 15.8% prevalence of severely prolonged QTc (> 500 ms) exceeds the 11.9% previously reported [3], possibly reflecting our older population (mean age 84.5 vs. ~80 years) and greater frailty and renal impairment.

To our knowledge, this is the first study evaluating the Tisdale score in a purely geriatric cohort, extending prior validation data from cardiac intensive care [5] and mixed cardiology/gastroenterology settings [7]. The score's predictive value persisted after adjustment for multimorbidity and renal dysfunction, supporting its applicability in older adults. Notably, despite higher Tisdale scores, females showed no greater QTc prolongation than males, possibly due to post‐menopausal loss of estrogen's protective effect on ventricular repolarization, survivor bias, or differential medication effects.

These findings support systematic Tisdale score calculation at admission to guide ECG monitoring intensity, medication review, and electrolyte management [8, 9]. The high prevalence of PPIs (48.4%), frequently prescribed without ongoing indication [10], represents a concrete deprescribing opportunity. Similarly, careful review of antidepressants, antipsychotics, and antiarrhythmics may identify candidates for dose reduction or discontinuation. In patients receiving loop diuretics—nearly half our cohort—regular potassium and magnesium monitoring with proactive repletion should be routine. Limitations include cross‐sectional design (precluding outcome data), single‐center setting, and absence of magnesium measurements.

## 5 Conclusion

QTc prolongation is highly prevalent in hospitalized older adults, and the Tisdale risk score provides robust, independent risk stratification in this population. Systematic score calculation at admission, combined with targeted medication review—particularly deprescribing of unnecessary PPIs and electrolyte monitoring in patients on diuretics—may reduce the burden of preventable arrhythmic events in geriatric inpatients.

## Author Contributions

F.F., M.Z.: study concept and design; S.F., L.G., A.G.: data acquisition; F.F., M.Z., G.T., R.M.: analysis and interpretation; F.F., A.G.: manuscript drafting; M.Z., F.F.: critical revision; R.M.: statistical analysis; M.Z.: funding; M.Z., G.T., F.F.: study supervision.

## Funding

This work was supported by Fondazione Cariverona.

## Conflicts of Interest

The authors declare no conflicts of interest.

## Supporting information


**Figure S1:** Distribution of patients by sex according to QT interval duration. Categories include: normal QTc, prolonged but ≤ 500 ms, and high‐risk QTc for TdP (> 500 ms).
**Figure S2:** Curve describing the probability of having a markedly prolonged QTc (> 500 ms) with increasing Tisdale score.
