# Supplementary material for: QTc Interval Prolongation and Associated Drug Therapy in Hospitalized Older Adults
Source: J Am Geriatr Soc. 2026 May 2;74(8):2445–8. doi: 10.1111/jgs.70477 (PMC13496369; doi:10.1111/jgs.70477)

Figure S1

Distribution of patients by sex according to QT interval duration. Categories include: normal QTc, prolonged but ≤500 ms, and high-risk QTc for TdP (>500 ms).

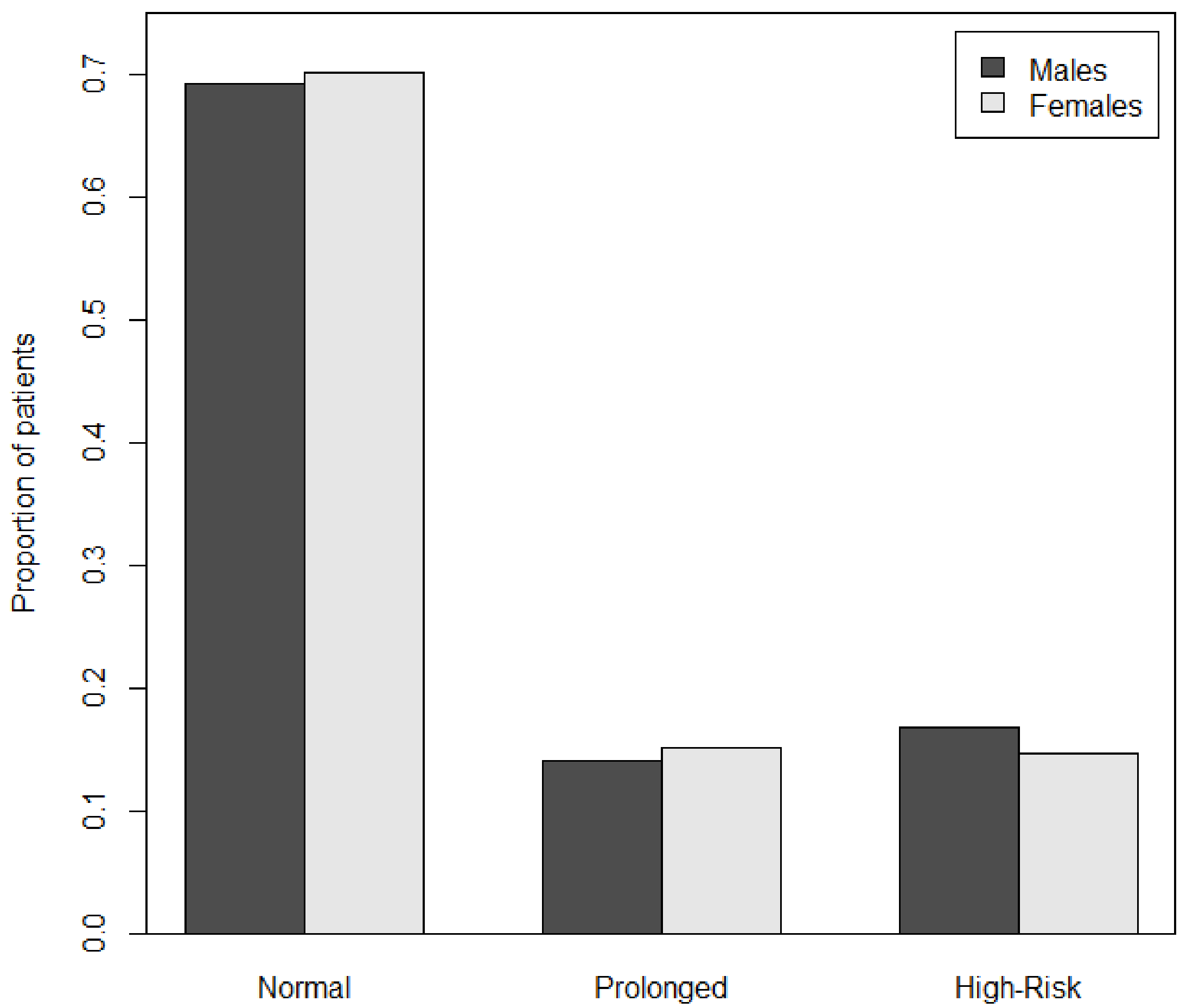

Figure S2
Curve describing the probability of having a markedly prolonged QTc (>500 ms) with increasing Tisdale score.

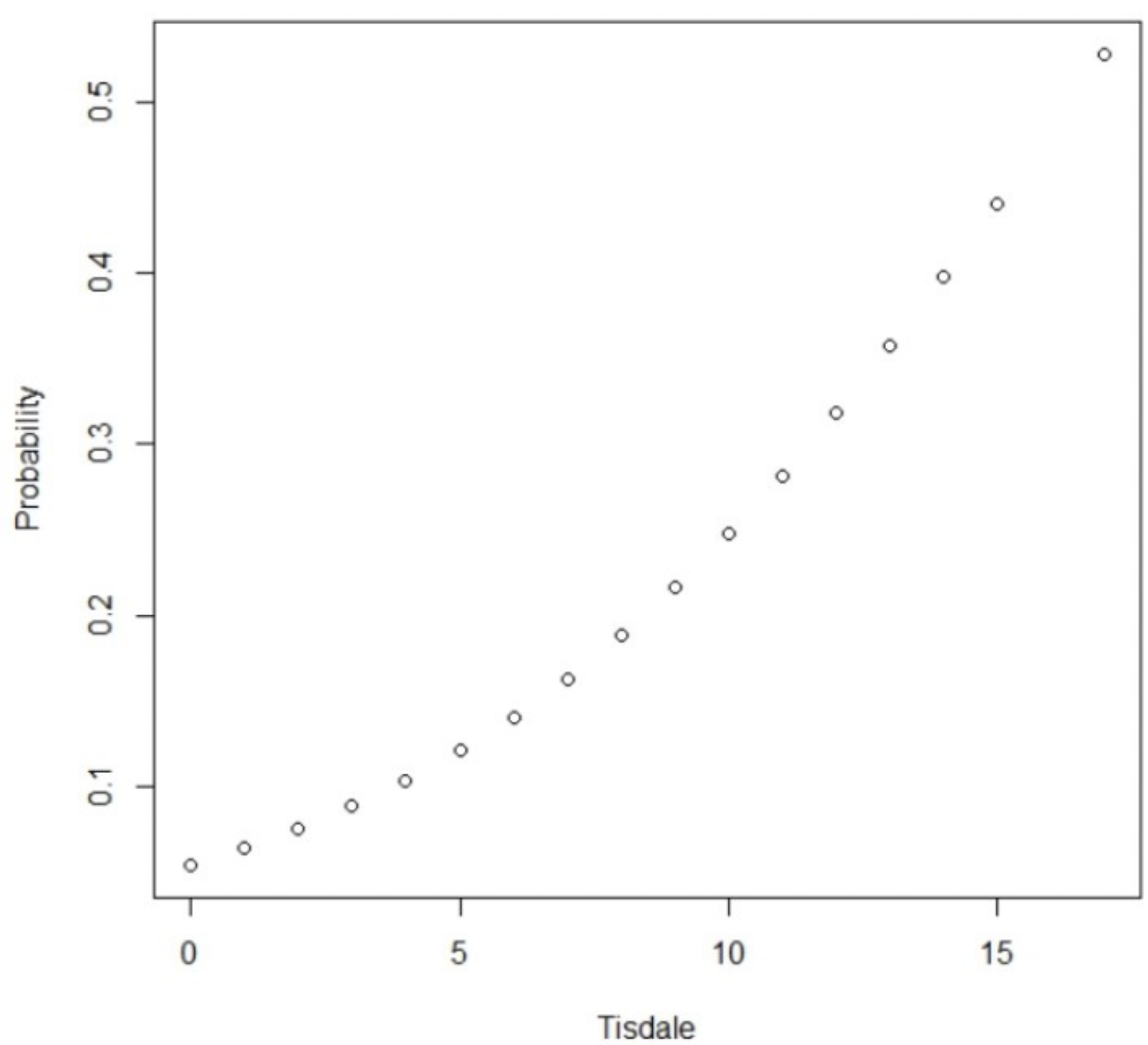

Supplement: Supplementary file 1 [file JGS-74-2445-s001.pdf]
